# A polygenic biomarker to identify patients with severe hypercholesterolemia of polygenic origin

**DOI:** 10.1002/mgg3.1248

**Published:** 2020-04-19

**Authors:** Luis G. Leal, Clive Hoggart, Marjo‐Riitta Jarvelin, Karl‐Heinz Herzig, Michael J. E. Sternberg, Alessia David

**Affiliations:** ^1^ Department of Life Sciences Centre for Integrative Systems Biology and Bioinformatics Imperial College London London United Kingdom; ^2^ Department of Medicine Imperial College London London United Kingdom; ^3^ Faculty of Medicine Center for Life Course Health Research University of Oulu Oulu Finland; ^4^ Biocenter Oulu University of Oulu Oulu Finland; ^5^ Unit of Primary Health Care Oulu University Hospital Oulu Finland; ^6^ Department of Epidemiology and Biostatistics MRC‐PHE Centre for Environment and Health School of Public Health Imperial College London London United Kingdom; ^7^ Department of Life Sciences College of Health and Life Sciences Brunel University London Middlesex United Kingdom; ^8^ Research Unit of Biomedicine Oulu University, Oulu Oulu University Hospital and Medical Research Center Oulu Oulu Finland; ^9^ Department of Gastroenterology and Metabolism Poznan University of Medical Sciences Poznan Poland

**Keywords:** hypercholesterolemia, lipids, polygenic risk score, risk stratification

## Abstract

**Background:**

Severe hypercholesterolemia (HC, LDL‐C > 4.9 mmol/L) affects over 30 million people worldwide. In this study, we validated a new polygenic risk score (PRS) for LDL‐C.

**Methods:**

Summary statistics from the Global Lipid Genome Consortium and genotype data from two large populations were used.

**Results:**

A 36‐SNP PRS was generated using data for 2,197 white Americans. In a replication cohort of 4,787 Finns, the PRS was strongly associated with the LDL‐C trait and explained 8% of its variability (*p* = 10^–41^). After risk categorization, the risk of having HC was higher in the high‐ versus low‐risk group (RR = 4.17, *p* < 1 × 10^−7^). Compared to a 12‐SNP LDL‐C raising score (currently used in the United Kingdom), the PRS explained more LDL‐C variability (8% vs. 6%). Among Finns with severe HC, 53% (66/124) versus 44% (55/124) were classified as high risk by the PRS and LDL‐C raising score, respectively. Moreover, 54% of individuals with severe HC defined as low risk by the LDL‐C raising score were reclassified to intermediate or high risk by the new PRS.

**Conclusion:**

The new PRS has a better predictive role in identifying HC of polygenic origin compared to the currently available method and can better stratify patients into diagnostic and therapeutic algorithms.

## Graphical abstract

Severe hypercholesterolemia (HC, LDL‐C > 4.9 mmol/l) affects over 30 million people worldwide. In this study, we generated a new polygenic risk score (PRS) for LDL‐C in 2,197 white Americans and validated our results in a replication cohort of 4,787 Finns. This new PRS explains 30% more LDL‐C variability (8% vs. 6%) and has a better predictive role in identifying HC of polygenic origin compared to existing methods.

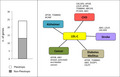

## 1 INTRODUCTION

Hypercholesterolemia is one of the most common conditions encountered in medical practice, as well as a known and, most crucially, modifiable cardiovascular risk factor. Severe hypercholesterolemia (HC) is defined as low‐density lipoprotein cholesterol (LDL‐C)> 4.9 mmol/L (>190 mg/dl) and is estimated to affect 14–35 million people worldwide (Sniderman, Tsimikas, & Fazio, 2014). Familial hypercholesterolemia (FH) is the most common cause of severe HC, with a prevalence of 1 in 250 individuals (Nordestgaard et al., 2013), affecting approximately 10 million individuals worldwide. If untreated, FH is associated with a 20‐fold increase in premature cardiovascular disease (CVD), with coronary events occurring in approximately 30% of women before the age of 60 years, and 50% of men by the age of 50 years (Nordestgaard et al., 2013). A monogenic origin of FH is confirmed in only 40% of patients with a clinical diagnosis of FH (Sharifi, Futema, Futema, Nair, & Humphries, 2017). In more than 90% of these genetically confirmed patients, a pathogenic heterozygous dominant mutation in the LDL receptor gene (*LDLR)* is detected, with recessive mutations in *APOB* and *PCSK9* present in the remainder (Berberich & Hegele, 2019). In 2013, Talmud et al. developed a weighted 12‐single nucleotide polymorphism (SNP) LDL‐C raising score validated in a white British population and suggested that in > 50% of patients with a clinical diagnosis of FH and negative genetic testing the origin of HC may be polygenic (Futema et al., 2015; Talmud et al., 2013). However, the utility of this score in clinical practice remains to be established and it is currently not incorporated in the NICE guidelines. In the last decade, several other LDL‐C polygenic scores have been proposed (Dron & Hegele, 2018). However, the major limitation of these studies is that, in the majority of cases, these association scores were developed in a specific population of individuals but results not replicated in a validation cohort.

The clinical management of patients with severe HC remains aggressive lipid lowering treatment guided by the patient's clinical history (Catapano et al., 2016; Sniderman et al., 2014). However, the finding that severe HC in a large percentage of patients meeting the clinical criteria for FH may be of polygenic rather than monogenic origin opens new questions on whether polygenic HC is a different phenotype compared to monogenic FH, thus, requiring different disease risk stratification algorithms for affected patients and their blood‐related family members. In order to answer these questions, it is mandatory to generate a polygenic biomarker with good accuracy and replicability in identifying patients with severe HC of polygenic origin. Moreover, such a polygenic marker would help stratify those patients in which DNA analysis reveals HC of neither monogenic nor polygenic origin. In such patients new, yet unidentified genes responsible for FH could be present (Futema, Bourbon, Williams, & Humphries, 2018).

Polygenic risk scores (PRSs) have gained wide interest in recent years as they may help deliver personalized medicine. PRSs have been used to identify patients at risk of several conditions, including cardiovascular (Inouye et al., 2018) and Alzheimer's disease (Chasioti, Yan, Nho, & Saykin, 2019).

The primary aim of this study was to develop an improved polygenic biomarker by generating an LDL‐C PRS. The score was obtained on a target cohort of white Americans using SNP summary data from the Global Lipid Genetics Consortium (GLGC), followed by validation on a second cohort of European Finnish individuals. We also compared the performance of the new PRS against the 12‐SNP LDL‐C raising score by Talmud et al. (2013) (which is currently used in the UK clinical setting) with a focus on reclassifying individuals with severe HC, who were deemed to be at a low risk of HC of polygenic origin.

## 2 MATERIALS AND METHODS

### 2.1 Ethical compliance

All participants to NFBC gave written informed consents and the Ethics Committee of Northern Ostrobothnia hospital district and the University of Oulu (Finland) approved the study. Protocols for the eMERGE network were approved by the Institutional Review Boards (IRBs) at the institutions where participants were recruited; all included participants provided written informed consent prior to inclusion in the study.

### 2.2 Populations

A study cohort of 2,764 white American individuals was obtained from The Electronic Medical Records and Genomics network (eMERGE, dbGaP Study Accession: phs000360.v3.p1) (McCarty et al., 2011). A replication cohort of 5,402 Finnish individuals was retrieved from the Northern Finland Birth Cohort 1966 (NFBC1966) (Järvelin et al., 2004; Sabatti et al., 2009). After data quality checking and genotype data imputation Haplotype Reference Consortium panel (McCarthy et al., 2016), the cohorts comprised 2,197 white Americans and 4,787 Finnish individuals of 39,131,578 genotyped and imputed SNPs (See details of data preprocessing in Supplementary Methods). Biochemical data were available for all subjects.

### 2.3 Construction of the PRS

The PRS of an individual *j* was defined by the weighted sum of LDL‐C raising alleles and depends on the set of *n* SNPs, the estimated SNP effect sizes (beta coefficients, *β_i_*) and the allele dosage carried by the individual (*x_i,j_*) according to the formula:

$${\text{PRS}}_{j}=\sum_{i=1}^{n}\beta_{i}\cdot x_{i,j}$$

The PRSice (Euesden, Lewis, & O’Reilly, 2015) algorithm was implemented as follows. First, genome‐wide summary statistics for SNPs associated with the LDL‐C trait (*p* < 1 × 10^−3^) were retrieved from the Global Lipid Genetics Consortium (GLGC). This initial set of SNPs was reduced by performing linkage disequilibrium (LD) pruning, thus retaining only the most significant SNPs in each LD block. Different LD thresholds (r^2^ between 0.1 and 0.8) were tested (detailed in Figure S1).

Sets of SNPs were defined over a range of *p*‐value thresholds (1 × 10^−3^ – 1 × 10^−100^) and evaluated by PRSice to identify the best PRS, that is, the one that maximizes the explained phenotypic variance in the white American cohort. At each *p*‐value threshold, the PRS was incorporated in a linear regression model to explain the LDL‐C continuous trait, while adjusting for the following covariates: age, gender, BMI, and ancestry differences captured by the first two components from multidimensional scaling. From each regression model, an incremental *R* ^2^ was computed by PRSice and plotted against the *p*‐value threshold. This *R* ^2^ is reported as the difference between the R^2^ of the full regression model (LDL‐C∼PRS + covariates) and the *R* ^2^ of the null model (LDL‐C∼covariates). The best PRS was the one achieving the highest *R* ^2^.

### 2.4 Performance assessment & statistical analysis

The PRS was assessed using the following statistical approaches:

- Model fit: A multiple linear regression model for the LDL‐C continuous trait was fitted and the R^2^ of the models compared. These *R* ^2^ values were calculated following the same approach described for PRSice (Euesden et al., 2015) (see Supplementary Material).
- Area under the curve (AUC): The phenotype was categorized in severe HC (LDL‐C > 4.9 mmol/L), intermediate HC (3.0 ≤ LDL‐C ≤ 4.9 mmol/L), and normal LDL‐C levels (LDL‐C < 3.0 mmol/L), and the classification accuracy of the scores was assessed by receiver operating characteristic (ROC) curves. We used the DeLong test to compare AUCs from different PRSs.
- The PRS was categorized using the deciles of the distribution: low‐risk (decile third and below), intermediate risk (deciles fourth, fifth, and sixth), high‐risk category (deciles seventh and above). This mirrors the score categorization used in the SNP LDL‐C raising score by Talmud et al. (2013), thus allowing comparison between methods. Afterwards, the difference in median LDL‐C levels across different PRS categories was tested using the Wilcoxon test. The risk ratio of having abnormal LDL‐C was calculated for the high‐risk category (relative to the low‐risk category) (see Supplementary Methods and Table S1). The same cutoffs for risk categorization identified in the American cohort were then applied to the Finnish cohort.
- The distribution of subjects with severe HC was analyzed across PRS categories. We compared the percentage of patients with severe HC in the low‐risk PRS category.

The list of genes (gene name, OMIM (MIM), and GenBank (RefSeq) identifiers) included in the PRS is presented in Table S2.

## 3 RESULTS

A cohort of 2,197 white American individuals was used to construct the PRS for the LDL‐C trait. The clinical and biochemical characteristics of the individuals included in the study are presented in Table 1 and Table S3.

**Table 1:** Clinical and biochemical characteristics of 2,197 white Americans from eMerge and of 4,787 Finns from the Northern Finland Birth Cohort (NFBC)

	American *n* = 2,197	Finnish *n* = 4,784
Age (years)	60 ± 11.5	31 ± 0.2
Males *n*. (%)	1,074 (49%)	2,307(48%)
LDL‐C (mmol/L)	3.60 (3.08–4.11)	2.90 (2.40–3.50)
HDL‐C (mmol/L)	1.19 (1.01–1.45)	1.51 (1.28–1.78)
Tg (mmol/L)	1.32 (0.94–1.86)	0.97 (0.73–1.38)
Smokers *n*. (%)	682 (31%)	1728 (37%)
BMI (kg/m^2^)	27.8 (24.9–31.4)	23.9 (21.9–26.6)

Note

Continuous variables are presented as median (interquartile Q1 and Q3), except for age, which is expressed as mean ± *SD*.

Abbreviations: BMI, body mass indexHDL‐C, high‐density lipoprotein cholesterol; LDL‐C, low‐density lipoprotein cholesterol; Tg, triglycerides.

A total of 8,224 SNPs (LDL‐C trait association *p* < 1 × 10^−3^) were analyzed by the PRSice algorithm. In a regression model (see Figure S2), the optimal PRS included 36 SNPs. The LD threshold of *r* ^2^ < 0.1 provided the best model fit (Figure S1 and S2). All 36 SNPs had a reported association with the LDL‐C trait with a *p* < 1 × 10^−20^ in the GLGC, and are presented in Table 2. This novel score explained 8% of the trait variability (*p* = 10^–41^) in a multiple regression analysis, which adjusts for covariates.

**Table 2:** Characteristics of the 36 SNPs included in the polygenic risk score

SNP	Location	Allele A	Allele B	Effect size	Gene	Q1	*p*‐value	SO
rs629301	1:109,275,684	G	T	0.1736	*CELSR2*	Yes	1E−170	3_prime_UTR_variant
rs4420638	19:44,919,689	A	G	0.2153	*APOC1*	No	9E−147	downstream_gene_variant
rs6511720	19:11,091,630	T	G	0.2108	*LDLR*	Yes	4E−117	intron_variant
rs1367117	2:21,041,028	G	A	0.1307	*APOB*	Yes	4E−114	missense_variant
rs515135	2:21,063,185	T	C	0.1458	*‐*	No	3E−109	intergenic_variant
rs1531517	19:44,738,916	A	G	0.2482	*‐*	No	4E−99	regulatory_region_variant
rs395908	19:44,870,308	A	G	0.1512	*NECTIN2*	No	1E−89	intron_variant
rs7254892	19:44,886,339	A	G	0.4181	*TOMM40*	No	3E−89	upstream_gene_variant
rs12721109	19:44,943,964	A	G	0.452	*APOC4*	No	1E−72	intron_variant
rs10402271	19:44,825,957	T	G	0.0916	*BCAM*	No	6E−63	downstream_gene_variant
rs4299376	2:43,845,437	T	G	0.0812	*ABCG8*	Yes	2E−47	intron_variant
rs12916	5:75,360,714	T	C	0.0755	*HMGCR*	No	5E−45	3_prime_UTR_variant
rs6859	19:44,878,777	G	A	0.0775	*NECTIN2*	No	6E−37	intron_variant
rs5930	19:11,113,589	A	G	0.0649	*LDLR*	No	3E−33	synonymous_variant
rs4953023	2:43,846,861	A	G	0.1347	*ABCG8*	No	3E−33	intron_variant
rs405509	19:44,905,579	G	T	0.0754	*APOE*	No	1E−31	upstream_gene_variant
rs2287029	19:10,806,008	T	C	0.0786	*DNM2*	No	4E−31	intron_variant
rs6725189	2:20,996,129	T	G	0.0713	*AC115619.1*	No	5E−30	downstream_gene_variant
rs2980875	8:125,469,505	G	A	0.0578	*AC091114.1*	No	3E−29	intron_variant
rs2479409	1:55,038,977	A	G	0.0671	*PCSK9*	Yes	2E−28	upstream_gene_variant
rs11685356	2:20,974,287	C	T	0.0668	*‐*	No	2E−27	intergenic_variant
rs1529729	19:11,052,886	T	C	0.0533	*SMARCA4*	No	2E−25	intron_variant
rs6547409	2:20,967,337	T	C	0.1525	*‐*	No	3E−25	intergenic_variant
rs12127701	1:109,295,642	G	A	0.126	*MYBPHL*	No	1E−24	intron_variant
rs253412	5:75,660,016	A	G	0.0559	*ANKDD1B*	No	1E−24	intron_variant
rs16979372	19:44,692,043	G	T	0.1499	*AC243964.2*	No	4E−24	intron_variant
rs413582	1:109,308,504	C	T	0.0518	*MYBPHL*	No	2E−23	upstream_gene_variant
rs2000999	16:72,074,194	G	A	0.0636	*HPR*	No	2E−22	intron_variant
rs6882076	5:156,963,286	T	C	0.0536	*TIMD4*	No	2E−22	upstream_gene_variant
rs287227	1:55,190,402	T	G	0.0753	*USP24*	No	2E−22	intron_variant
rs10401969	19:19,296,909	T	C	0.106	*SUGP1*	No	7E−22	intron_variant
rs649129	9:133,278,860	T	C	0.0607	*ABO*	No	8E−22	upstream_gene_variant
rs174583	11:61,842,278	T	C	0.0511	*FADS2*	No	1E−21	intron_variant
rs1004165	19:44,728,939	G	A	0.0566	*RF00285*	No	4E−21	upstream_gene_variant
rs16996148	19:19,547,663	T	G	0.0877	*CILP2*	No	6E−21	downstream_gene_variant
rs10198175	2:20,934,123	G	A	0.0864	*—*	No	7E−21	intergenic_variant

Note

SNPs are presented using dbSNP Id (Sherry et al., 2001). The chromosome harboring the SNP and the SNP alleles (A and B) are shown. Chromosome location is according to GRCh38. Allele B is the LDL‐C rising allele. Effect size and *p*‐value for each SNP are according to GLGC. SO, is the Sequence Ontology term from Ensembl (Zerbino et al., 2018). Q1 indicates whether the SNP is used in the 12‐SNPs raising score by Talmud et al. (2013).

OMIM (MIM) and GenBank (RefSeq) identifiers (Id) are presented in Table S2.

After score categorization (Table 3), median LDL‐C values were significantly higher in the high‐risk (3.85 mmol/L, IQR: 3.34–4.36) versus the low‐risk category (3.36 mmol/L, IQR: 2.81–3.90; *p* = 10^–29^, Wilcoxon test). Individuals in the high category had a significantly higher risk of severe HC relative to individuals in the low category (RR = 4.1, CI: 2.2–7.4, *p* = 10^–5^).

**Table 3:** LDL‐C levels in the PRS risk classes in the white American (eMERGE) and Finnish (NFBC) populations

PRS risk	American *n* = 2,197	Finnish *n* = 4,784
Low		
Subjects, *n*	659	1,246
LDL‐C (mmol/L)	3.36 (1.09)	2.6 (1.00)
PRS score range	4.37–5.98	4.13–5.98
Intermediate		
Subjects, *n*	879	2,103
LDL‐C (mmol/L)	3.57 (0.96)	2.9 (1.10)
PRS score range	5.98–6.44	5.98–6.44
High		
Subjects, *n*	659	1,435
LDL‐C (mmol/L)	3.85 (1.02)	3.2 (1.20)
PRS score range	6.44–7.60	6.44–7.50

Note

LDL‐C levels are presented as median and interquartile range, IQR (calculated as Q3‐Q1).

### 3.1 LDL‐C PRS applied on the Northern Finland Birth Cohort (NFBC)

The new PRS was applied to a cohort of 4,787 Finnish individuals from the *Northern Finland Birth Cohort 1966 (*NFBC), whose clinical and biochemical data are presented in Table 1 and Table S3. In this replication cohort, our score explained 8% of LDL‐C variability. Moreover, after score categorization, the difference in median LDL‐C levels, as well as the risk of having severe HC, were significantly higher in the high‐ versus low‐genetic risk category (median LDL‐C: 3.2 mmol/L vs. 2.6 mmol/L, *p* = 10^–63^; RR = 4.8 (CI: 2.6–8.9), *p* = 10^–7^).

### 3.2 Comparison with the currently available method (SNP LDL‐C raising score)

We compared the results obtained with our new PRS to those obtained using the SNP LDL‐C raising score of Talmud et al. (2013) estimated in a white British cohort. Our PRS was more accurate compared to the SNP LDL‐C raising score in both the American (AUC 0.65 versus. 0.61, *p* = .12 DeLong test) and Finnish populations (AUC = 0.67 vs. 0.65, *p* = .36, DeLong test) and was able to explain 30% more of trait variance (8% vs. 6% in the American population and 8% vs. 6% in the Finnish population).

Afterwards, the categorized PRS and SNP LDL‐C raising score were compared. In the white American cohort, 45% (230/506) versus 42% (213/506) of individuals with normal level LDL‐C (<3 mmo/L) were classified in the low‐risk category, and 50% (53/107) versus. 46% (49/107) individuals with severe HC were classified in the high‐risk category using the PRS and SNP raising score, respectively.

In the Finnish cohort, which is a younger cohort compared to the Americans (mean age 31 yrs, *SD* 0.2 versus. 60 yrs, *SD* 11.5 in the Americans), with a healthier lipid profile (57% versus 23% individuals with LDL‐C levels below 3 mmol/L, Table S3), 32% (877/2733) of individuals with normal LDL‐C levels were classified as low risk by both methods. However, 53% (66/124) versus 44% (55/124) of individuals with severe HC were included in the high‐risk category using the PRS and SNP raising score, respectively (*p* = .16), thus confirming a trend toward a better performance of PRS versus SNP raising score in the replication cohort.

When results were analyzed by a subject‐to‐subject comparison, the two methods showed concordance in 37% of cases with severe HC in the American cohort (34 individuals classified in the high‐risk category by both methods and 6 individuals classified as low‐risk by both methods) and 42% of severe HC cases in the Finnish cohort (44 individuals classified as high risk by both methods and eight individuals classified as low risk by both methods).

### 3.3 Reclassification of patients with severe HC and low risk of HC with the SNP raising score

An important potential use of the PRS in the clinical setting is for clarifying the genetic background of patients with severe HC, especially individuals that meet the clinical criteria for FH but have no pathogenic variants in the genes responsible for the monogenic form of this condition, namely *LDLR*, *APOB,* or *PCSK9* (approximately 40% of patients who undergo genetic testing for FH) (Berberich & Hegele, 2019). These individuals are candidates for further extensive genetic testing to identify novel genetic causes of HC. In our study, 11% (26) individuals with severe HC (13 in the American and 13 in the Finnish population) were classified as low risk of HC by the SNP LDL‐C raising score. However, when using the new PRS, 46% of these individuals (7/13 in American and 5/13 in Finnish population) were reclassified to either the intermediate (11) or high‐risk category (1 individual), suggesting that the genetic makeup of these cases can, at least in part, explain their severe HC. Of note, none of the patients classified as low risk by PRS were classified as high risk by the SNP LDL‐C raising score. Six subjects with HC classified as intermediate risk of polygenic origin by the SNP LDL‐C raising score, were classified as low risk (third decile) by PRS.

### 3.4 Functional annotations and pleiotropy analysis of genes in the new LDL‐C PRS

The 36 SNPs in the new PRS map to 23 genes (Table S2), six of which (*PCSK9, APOB, LDLR, APOE, CELSR2,* and *ABCG8*) are also present in the SNP LDL‐C raising score (see Table 2). These 23 genes show a significant enrichment in Gene Ontology (GO) terms related to cholesterol homeostasis (p 1x10^‐4^) and lipoprotein processes (*p* = 7 × 10^−4^). No enrichment was found in KEGG metabolic pathways annotation, which suggests that multiple metabolic pathways may be implicated in the development of severe HC of polygenic origin.

In the new PRS, we did not include the two SNP (rs7412 and rs429358), which define the APOE *ε*2, *ε*3, and *ε*4 haplotype. However, these two SNP are in LD (LD > 0.35 between rs7412 and rs7254892 in *TOMM40* and between rs429358 and rs4420638 in APOC4) in both American and Finnish populations (see Figure S3).

**Figure 1:**
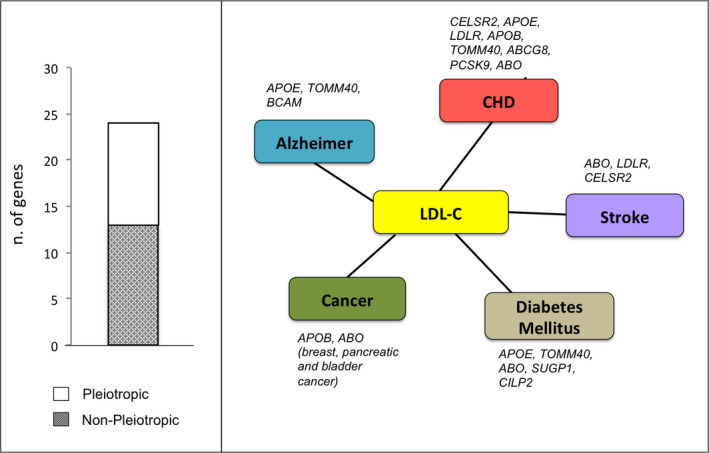
*Pleiotropic nature of the genes included in the PRS for LDL‐*C trait. 47.8% (11/23) of genes that harbor the SNPs included in the PRS are pleiotropic (left panel) and associated with coronary artery disease (CHD), Stroke, Alzheimer disease, cancer (breast, bladder, and pancreatic), and diabetes mellitus type 2 (right panel)

As severe HC is often associated with other cardiovascular risk factors and the degree of aggressive lipid lowering treatment is dictated by the patient overall cardiovascular risk, we assessed the pleiotropic nature of the 23 genes in the new PRS by examining their association with other traits in the GWAS catalog, using a cutoff for association *p* < 1 × 10^−8^. Eight genes (*CELSR2, APOE, LDLR, APOB, TOMM40, ABCG8, PCSK9,* and *ABO*) were associated with coronary artery disease, five with diabetes mellitus type 2 (*APOE, TOMM40, SUGP1, ABO,* and *CILP2*) and three with Alzheimer's disease (*BCAM, TOMM40*, and *APOE*) (see Table S4). Moreover, two genes (*APOB* and *ABO*) were reportedly associated with cancer (bladder, pancreatic, or breast) and three genes (*ABO, LDLR,* and *CELSR2*) with Stroke (Figure 1). Of 36 SNPs included in the PRS, 10 were also pleiotropic (or in LD (*r* ^2^ > .1) with variants reported for these traits). The list of pleiotropic SNPs is as follows: association with coronary artery disease (rs629301, rs4299376, rs649129, rs1529729, rs6511720, rs7254892, and rs4420638), stroke (rs629301, rs649129, rs1529729, and rs6511720), diabetes mellitus type 2 (rs649129, rs10401969, rs16996148, rs405509, and rs4420638), and Alzheimer's disease (rs7254892, rs405509, and rs4420638).

## 4 DISCUSSION

We constructed a new PRS for LDL‐C from an initial set of thousands of SNPs at GWAS *p*‐value threshold < 10^–3^ and demonstrated that it is robustly associated with the LDL‐C trait in two independent populations of white European ancestry from the United States of America and Finland. Compared to the existing 12‐SNP LDL‐C raising score (currently used in the setting of FH genetic testing by genetic laboratories in England, UK), the new PRS was able to explain 30% more of LDL‐C trait variability and to identify a polygenic risk component, therefore, reclassifying several patients otherwise deemed as low risk for hypercholesterolemia of polygenic origin. The PRS for LDL‐C can have several applications in clinical practice including i) identifying patients with a clinical diagnosis of FH who are at high likelihood of HC of polygenic origin, and ii) inclusion into algorithms for the early stratification of patients at risk of HC and other comorbidities, both cardiovascular and Alzheimer's disease, for early life style modifications.

The SNP LDL‐C raising score by Talmud et al. (2013), and the work published by the same group in patients with a clinical diagnosis of possible FH (Sharifi, Futema, Nair, & Humphries, 2019; Sharifi, Higginson, et al., 2017), suggests that HC of polygenic origin could be a new phenotype, distinct from monogenic FH, thus requiring a different clinical approach. However, the most informative SNP set should be used to identify FH patients without a confirmed monogenic diagnosis (FH/M‐) patients, and distinguish those with polygenic HC from those in which the genetic background of HC remains unexplained (10%–15% of cases) (Sharifi, Futema, et al., 2017). The same research group who developed the SNP LDL‐C raising score (which consists of 12 SNPs and is currently used by the Bristol genetics laboratories in the UK in the genetic screening of patients with a clinical diagnosis of FH), attempted to improve their score by manually choosing and adding 21 additional SNPs associated with the LDL‐C trait to their original 12‐SNP score. However, this did not result in a better diagnostic performance (Futema et al., 2015).

Although PRSs can be built using a small number (typically < 100) of SNPs at GWAS significant level (*p*‐value < 5 × 10^−8^), the field is now migrating toward the use of genome‐wide polygenic scores consisting of thousands of SNPs with higher *p*‐values (Goldstein, Yang, Salfati, & Assimes, 2015). These mega polygenic scores have the potential of being more informative compared to small ones (Natarajan et al., 2018); however, this comes at the cost of intensive computational analysis and no genome‐wide polygenic score is currently available for LDL‐C in clinical practice.

In this study, we used an unbiased method for selecting the most informative SNPs associated with LDL‐C from an initial set of over 8,000 SNPs, to construct a polygenic score for LDL‐C. Although the AUC for PRS was only marginally better compared to that of LDL‐C SNP score, the PRS was better in classifying patients into low or high risk compared to the LDL‐C SNP score method. However, this was just a trend possibly because of the small number of patients with severe HC in our two cohorts.

The 23 genes harboring the 36 SNPs selected for the PRS show enrichment in Gene Ontology terms related to lipid metabolism, thus further confirming the validity of the selection process. Pathways analysis did not show any enrichment, which suggests that small defects in multiple metabolic pathways may be involved in hypercholesterolemia of polygenic origin.

We are still far from understanding the genetic causes of FH, a condition associated with a 20‐fold increased risk of CHD compared to the general population (Nordestgaard et al., 2013). FH has an estimated prevalence of 1 in 250 individuals. In approximately 40% of FH patients an inherited pathogenic DNA point mutation (Sharifi, Futema, et al., 2017) (monogenic FH, FH/M+) is present, whereas in 50% of cases HC is deemed to be of polygenic origin. In the remaining 10% of cases, FH is of unknown origin. Mutations in yet unknown gene/s could be present in these patients with FH of unknown origin and pose a novel drug target for severe HC. Narrowing down the number of individuals with primary HC and no known pathogenetic cause (HC of monogenic or polygenic origin) is crucial for studies aimed at understanding the pathogenesis of HC. New improved scores, such as ours, that include novel LDL‐C SNPs can help identify, and hence reclassify, patients in whom HC of a polygenic origin is present, thus improving diagnostic and therapeutic algorithms.

We found that many of the genes included in the PRS have pleiotropic effects. We and others have noted that gene pleiotropy is common in genes implicated in both rare (Ittisoponpisan, Alhuzimi, Sternberg, & David, 2017) and common disorders (Price, Spencer, & Donnelly, 2015). In this study, 47.8% of the genes in the PRS were also associated with conditions, such as Alzheimer's, CHD, and diabetes in GWASs. Indeed, there is a well‐known association between HC and Alzheimer's (Park et al., 2013) or CHD (Humphries et al., 2019) disease. The PRS could, thus, be of help in identifying HC patients who are at risk of developing comorbidities, thus contributing toward achieving personalized medicine.

An important limitation of our study is the small number of patients with severe HC in the two cohorts. Future work will involve applying the PRS in FH patient cohorts and, in particular, to FH/M‐ patients. Moreover, it will be important to evaluate the correlation between polygenic risk for HC, as defined by the PRS, to the risk of cardiovascular events.

In conclusion, we developed a polygenic biomarker based on 36 SNPs that is able to identify patients at an increased risk of HC and associated comorbidities as a result of their genetic makeup.

## CONFLICT OF INTEREST

The authors declare that there is no conflict of interest.

## AUTHOR CONTRIBUTION

AD and LGL conceptualized the study and contributed to the first draft of the manuscript. LGL performed the molecular analyses. AD contributed to the bioinformatic analyses. AD, CH, and MJS contributed to interpretation of bioinformatic data. All authors critically reviewed the manuscript.

## Supporting information

Supinfo
