# Supplementary material for: A polygenic biomarker to identify patients with severe hypercholesterolemia of polygenic origin
Source: Mol Genet Genomic Med. 2020 Apr 19;8(6):e1248. doi: 10.1002/mgg3.1248 (PMC7284038; doi:10.1002/mgg3.1248)
Supplement: Supplementary file 1 — Supinfo [file MGG3-8-e1248-s001.doc]

**Supplemental Material**

**A polygenic biomarker to identify patients with severe hypercholesterolemia of polygenic origin**

Luis G. Leal, Clive Hoggart, Marjo-Riitta Jarvelin, Karl-Heinz Herzig, Michael JE Sternberg, Alessia David

**Supplementary Methods**

***Study cohorts***

An initial cohort of 2,764 American individuals of self-reported white ethnicity was retrieved from the Electronic Medical Records and Genomics network (eMERGE, dbGaP Study Accession: phs000360.v3.p1). Their lipid measurements come from a longitudinal panel studied by the Northwestern University (dbGaP Document Accession: phd003541.1). These measurements are not biased by comorbidities such as type 2 diabetes medications, hormone replacement therapies or antilipemic drugs. Since ancestry data are self-reported, genotype data were analysed against the 1000 Genomes Project, and individuals that did not cluster with any European population were considered of wrong self-reported ancestry and removed from the final dataset. The final cohort included 2,197 individuals.

***Replication cohort***

To replicate the results of the best formulation, a second group of 5,402 white individuals from the Northern Finland Birth Cohort 1966 (NFBC1966) (Järvelin et al., 2004), (Sabatti et al., 2009) was used. The lipid profile of each individual was obtained on fasting samples and subjects on diabetic medications were removed. In this cohort there was no evidence of any treatment with antilipemic medications. The final cohort included 4,787 individuals.

***Genotyping data imputation***

The imputation of both cohorts (eMERGE and NFBC1966) was conducted on the Michigan Institute Servers2 (Das et al., 2016) using the Eagle algorithm (Loh et al., 2016) and the Haplotype Reference Consortium panel (McCarthy et al., 2016) (version 1.1) and allowed expanding the genotyping coverage to 39,131,578 SNPs.

***Poligenic Risk Score (PRS) calculation***

The PRSice (Euesden et al., 2015) algorithm was used to calculate the best PRS. PRSice selects SNPs at different GWAS p-values, test for evidence of association between the PRS and LDL-C trait, and identifies the p-value threshold for the most predictive set of variants, which will form the final PRS.

Beta coefficients were retrieved from the Global Lipids Genetics Consortium (GLGC). When the reported effect size was negative, the absolute effect size was computed and the second allele was taken as the risk allele.

To assure that SNPs effects can be summed up in the PRS, a clumping step was performed by PRSice to remove non-independent SNP effects. First, groups of SNPs in high linkage disequilibrium (LD) were identified (r2 < 0.1) within regions of 250 kb. The SNP with the lowest p-value in the group was selected as a proxy SNP, so the remaining SNPs in high LD were removed. The performance of PRS was tested under different clumping LD thresholds (r^2^ ranging from 0.1 to 0.8) (Figure S2 panel A and B)

The R^2^ calculated by PRSice is a goodness-of-fit measure to estimate the proportion of variance explained by the PRS. This is reported by PRSice as the difference between the R^2^  of the full regression model (LDL-C ~ PRS + covariates) and the R^2^ of the null model (LDL-C ~ covariates).

***Risk Ratio***

The Risk Ratio (RR) of severe HC in the High risk category (relative to the Low risk category) is calculated with the entries of Table S1.

(Equation S1)

**Table S1. Contingency table for the PRS and LDL-C categories in Equation S1.**

| **Subjects** | **LDL-C > 4.9 mmol/L** | **LDL-C ≤ 4.9 mmol/L** |
| --- | --- | --- |
| **High Risk** | a | b |
| **Low Risk** | c | d |

**Table S2 OMIM (MIM) and GenBank (RefSeq) identifiers (Id) are presented for the genes harbouring the SNP included in the PRS. Gene names are the HGNC approved gene symbol.**

| **Gene name** | **MIM Id** | **RefSeq Id** |
| --- | --- | --- |
| *ABCG8* | 605460 | NC_000002.12 |
| *ABO* | 110300 | NC_000009.12 |
| *ANKDD1B* | n.a. | NC_000005.10 |
| *APOB* | 107730 | NC_000002.12 |
| *APOC1* | 107710 | NC_000019.10 |
| *APOC4* | 600745 | NC_000019.10 |
| *APOE* | 107741 | NC_000019.10 |
| *BCAM* | 612773 | NC_000019.10 |
| *CELSR2* | 604265 | NC_000001.11 |
| *CILP2* | 612419 | NC_000019.10 |
| *DNM2* | 602378 | NC_000019.10 |
| *FADS2* | 606149 | NC_000011.10 |
| *HMGCR* | 142910 | NC_000005.10 |
| *HPR* | 140210 | NC_000016.10 |
| *LDLR* | 606945 | NC_000019.10 |
| *MYBPHL* | n.a. | NC_000001.11 |
| *NECTIN2* | 600798 | NC_000019.10 |
| *PCSK9* | 607786 | NC_000001.11 |
| *SMARCA4* | 603254 | NC_000019.10 |
| *SUGP1* | 607992 | NC_000019.10 |
| *TIMD4* | 610096 | NC_000005.10 |
| *TOMM40* | 608061 | NC_000019.10 |
| *USP24* | 610569 | NC_000001.11 |

n.a., not available

**Table S3** **Categorized LDL-C levels in the two population cohorts: 2,197 white American individuals from eMerge and of 4,787 Finnish individuals from the Northern Finland Birth Cohort 1966 (NFBC1966).**

**Table S4 Genes in the new PRS which harbour SNPs reported to have an association (AS) with Coronary Artery disease, Diabetes mellitus type 2 or Alzheimer’s disease in the Genome wide Association catalog (GWAS). Data are presented at two GWAS p-value cutoffs of p< 1x10^-6^ and p < 1x10^-8^ .**

|  | Coronary Artery disease | | Diabetes mellitus type 2 | | Alzheimer’s disease | |
| --- | --- | --- | --- | --- | --- | --- |
| Gene | p < 10^-6^ | p < 10^-8^ | p < 10^-6^ | p < 10^-8^ | p < 10^-6^ | p < 10^-8^ |
| *CELSR2* | + | + |  |  |  |  |
| *APOE* | + | + | + | + | + | + |
| *LDLR* | + | + |  |  | + |  |
| *APOB* | + | + |  |  |  |  |
| *TOMM40* | + | + | + | + | + | + |
| *ABCG8* | + | + |  |  |  |  |
| *HMGCR* | + |  |  |  |  |  |
| *PCSK9* | + | + |  |  |  |  |
| *ABO* | + | + | + | + |  |  |
| *SUGP1* |  |  | + | + |  |  |
| *CILP2* |  |  | + | + |  |  |
| *DMM2* |  |  |  |  | + |  |
| *BCAM* |  |  |  |  | + | + |

+, Association reported in GWAS catalog

**Figure S1** Selection of the optimal LD threshold for the SNV clumping step in PRSice. Panel A: R^2^ as a function of the LD threshold. Panel B: Number of SNVs in the PRS as a function of the LD threshold.


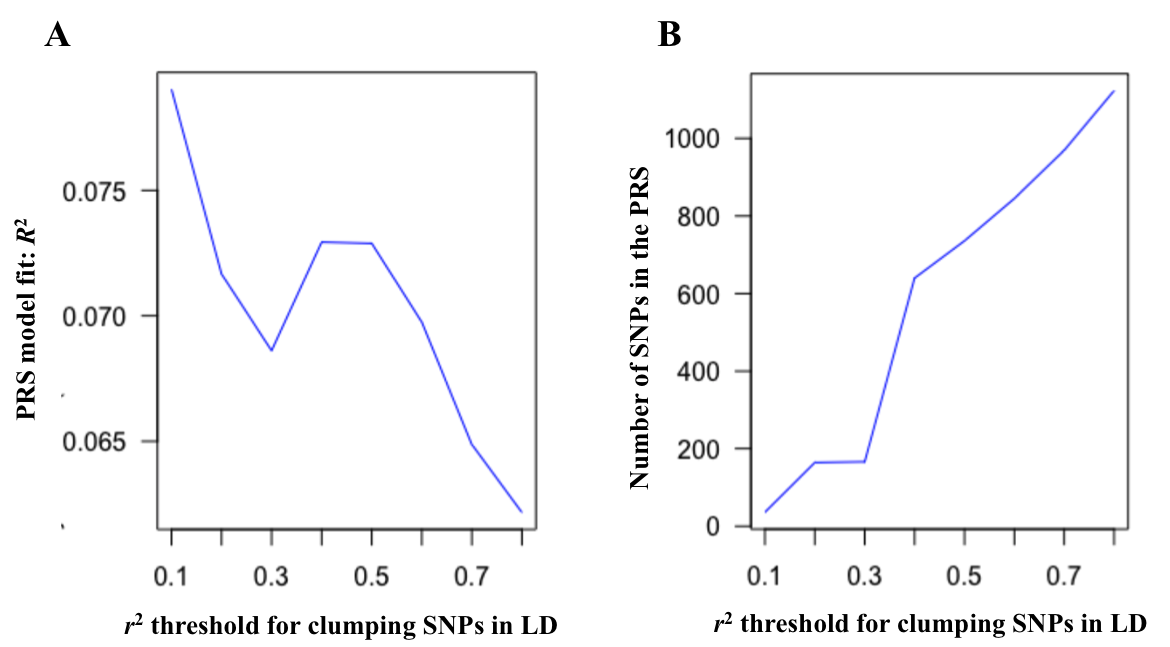


**Figure S2 PRS model fit using the PRSice algorithm**.

Panel A: Barplot generated by the PRS algorithm. The *R^2^* is shown over a range of GWAS p-values thresholds. This *R^2^* is the difference between the *R^2^* in the full regression model (LDL-C ~ PRS plus covariates) and the *R^2^* in the null model (LDL-C ~ covariates). The best model was found at p<10^-20^ (maximum R^2^=0.078). The bars color corresponds to the p-values for association between each PRS and LDL-C levels in the null regression model. Panel B: Number of SNPs in the PRS at each GWAS p-value threshold. The best model contains 36 SNPs.

**Figure S3** **Linkage disequilibrium (LD) matrix for chromosome 19**.

The LD is calculated for SNPs in PRS (formulation 1) and LDL SNP raising score (formulation 2). Results are presented for (A) white American cohort (eMERGE) and (B) Finnish cohort (NFBC).

**References**

Das S, Forer L, Schönherr S, Sidore C, Locke AE, Kwong A, Vrieze SI, Chew EY, Levy S, McGue M, Schlessinger D, Stambolian D, et al. 2016. Next-generation genotype imputation service and methods. Nat Genet 48:1284–1287.

Euesden J, Lewis CM, O’Reilly PF. 2015. PRSice: Polygenic Risk Score software. Bioinformatics 31:1466–1468.

Järvelin M-R, Sovio U, King V, Lauren L, Xu B, McCarthy MI, Hartikainen A-L, Laitinen J, Zitting P, Rantakallio P, Elliott P. 2004. Early life factors and blood pressure at age 31 years in the 1966 northern Finland birth cohort. Hypertension 44:838–846.

Loh P-R, Danecek P, Palamara PF, Fuchsberger C, A Reshef Y, K Finucane H, Schoenherr S, Forer L, McCarthy S, Abecasis GR, Durbin R, L Price A. 2016. Reference-based phasing using the Haplotype Reference Consortium panel. Nat Genet 48:1443–1448.

McCarthy S, Das S, Kretzschmar W, Delaneau O, Wood AR, Teumer A, Kang HM, Fuchsberger C, Danecek P, Sharp K, Luo Y, Sidore C, et al. 2016. A reference panel of 64,976 haplotypes for genotype imputation. Nat Genet 48:1279–1283.

Sabatti C, Service SK, Hartikainen A-L, Pouta A, Ripatti S, Brodsky J, Jones CG, Zaitlen NA, Varilo T, Kaakinen M, Sovio U, Ruokonen A, et al. 2009. Genome-wide association analysis of metabolic traits in a birth cohort from a founder population. Nat Genet 41:35–46.
